# Reduced Connexin 43 expression is associated with tumor malignant behaviors and biochemical recurrence-free survival of prostate cancer

**DOI:** 10.18632/oncotarget.11231

**Published:** 2016-08-11

**Authors:** Ning Xu, Hui-Jun Chen, Shao-Hao Chen, Xue-Yi Xue, Hong Chen, Qing-Shui Zheng, Yong Wei, Xiao-Dong Li, Jin-Bei Huang, Hai Cai, Xiong-Lin Sun

**Affiliations:** ^1^ Department of Urology, The First Affiliated Hospital of Fujian Medical University, Fuzhou, China; ^2^ Department of Pathology, The First Affiliated Hospital of Fujian Medical University, Fuzhou, China

**Keywords:** Connexin 43, prostate cancer, biochemical recurrence, immunohistochemistry, malignant behavior

## Abstract

Connexin 43, a gap junction protein, coordinates cell-to-cell communication and adhesion. Altered Connexin 43 expression associated with cancer development and progression. In this study, we assessed Connexin 43 expression for association with clinicopathological features and biochemical recurrence of prostate cancer after radical prostatectomy. Pathological specimens were collected from 243 patients who underwent radical prostatectomy and from 60 benign prostatic hyperplasia (BPH) patients to construct tissue microarrays and immunohistochemical analysis of Connexin 43 expression. Kaplan-Meier curves and multivariable Cox proportion hazard model were performed to associate Connexin 43 expression with postoperative biochemical recurrence-free survival (BFS). Connexin 43 expression was significantly reduced or lost in tumor tissues compared to that of BPHs (39.1% vs. 96.7%, *P*<0.001). Reduced Connexin 43 expression was associated with high levels of preoperative PSA, high Gleason score, advanced pT stage, positive surgical margin, extracapsular extension, and seminal vesicle invasion (*P* < 0.05, for all). Kaplan–Meier curves showed that reduced Connexin 43 expression was associated with shortened postoperative BFS (*P* < 0.001). Multivariate analysis showed that reduced Connexin 43 expression, high Gleason score and advanced pT stage were independent predictors for BFS of patients (*P <* 0.05). Connexin 43 expression was significantly reduced or lost in prostate cancer tissues, which was associated with advanced clinicopathological features and poor BFS of patients after radical prostatectomy.

## INTRODUCTION

Prostate cancer morbidity and mortality are rising in numbers of developing countries, including China, although advancement in treatment options and/or early detection significantly reduced prostate cancer mortality in developed countries [1]. Appropriate prostate cancer staging and grading will guide treatment selections and predict prognosis of prostate cancer [1, 2]. Serum prostate-specific antigen (PSA) level, pT stage, and Gleason score were traditionally recognized as prognostic factors, although their accuracy may have limitations [3–5]. To date, radical prostatectomy is one option to cure prostate cancer patients, but certain percentage of such patients still develop a biochemical recurrence in early postoperative period of time [6]. The biochemical recurrence after radical prostatectomy was defined as two consecutive rising of serum PSA levels greater than 0.2 ng/ml during the follow-up [7]. Thus, research on identification and evaluation of novel molecular markers could help urologists to precisely assess prostate cancer risk, recurrence, and prognosis clinically [8, 9].

Connexin 43 is a gap junction (GJ) protein to form a transmembrane protein channel structure between cells and functions to promote the material exchange and communication of adjacent cells, which plays an important role in regulation of cell proliferation, differentiation, and homeostasis [10, 11]. A previous study showed that Connexin 43 expression was decreased or lost in prostate cancer tissues, which was significantly associated with disease progression and unfavorable prognosis [12]. Expecting to provide insightful information regarding Connexin 43 expression as a biomarker for prostate cancer progression, we collected pathological specimens from 243 patients who underwent radical prostatectomy and from 60 benign prostatic hyperplasia (BPH) patients for tissue microarray construction and immunohistochemical analysis of Connexin 43 expression. We then associated Connexin 43 expression with clinicopathological characteristics or biochemical recurrence after surgery.

## MATERIALS AND METHODS

### Tissue specimen and data collection

This study was approved by The ethics committee of the First Affiliated Hospital of Fujian Medical University (Fuzhou, China). After patients provided the informed consent, we collected pathological specimens from 243 patients who underwent radical prostatectomy between January 2005 and January 2010 and from 60 benign prostatic hyperplasia (BPH) patients between January 2005 and January 2010 at our hospital. Patients with detectable PSA (>0.1 ng/mL) at the first month after radical prostatectomy usually have had persistent cancer and received postoperative radiotherapy and/or hormone therapy, so such patients were excluded from this study [13, 14]. Patients were diagnosed histologically and didn't receive any preoperatively adjuvant endocrine therapy or radiotherapy and patients with uncompleted clinical and pathological data were excluded from this study. All prostate cancer cases were staged according to clinical staging of prostate cancer, including digital rectal examination, transrectal ultrasonography, serum PSA and bone scans, while some cases presented with bilateral lymphadenectomy and had showed no lymph node metastasis. After radical prostatectomy, patients were followed up regularly, i.e., clinical examination and serum PSA test at the first month after surgery, then every 3 months for two years, and semiannually thereafter. The time of biochemical recurrence was recorded or to the last follow-up date (November 2015). Patients were followed up for 61 months (median, ranged between 7 and 97 months) and 35 of 243 (14.4%) patients had biochemical disease progression and the median time of biochemical recurrence was 37 months (ranged between 10 and 73 months). These 60 cases of BPH were randomly selected from the same hospital as a control. Clinical data, including age, preoperative PSA, prostate volume, PSA density (PSAD), body mass index (BMI), percentage of positive biopsies, pathological T stage, positive surgical margin, extracapsular extension and seminal vesicle invasion were also collected (Table 1).

**Table 1:** Clinicopathological features of 243 prostate cancer patients

Variables	Mean or Median ± SD (Range) or *n* (%)
Age (years)	
Mean ± SD	68.00 ± 7.04
Range	46 -79
BMI (kg/m^2^)	
Mean ± SD	25.42 ±4.40
Range	17.02 - 33.25
Prostate volume (cm^3^)	
Mean ± SD	38.23 ± 10.21
Range	23-57
PSA level (ng/ml)	
Mean ± SD	13.99 ± 10.21
Range	2.02-59.10
PSAD (ng/ml·cm^3^)	
Mean ± SD	0.40 ± 0.41
Range	0.05 - 2.17
Percentage of positive biopsies [n (%)]	
< 50	166 (68.3)
≥ 50	77 (31.7)
Pathological stage (pT) [n (%)]	
T1	89 (36.6)
T2	130 (53.5)
T3	24 (9.9)
Gleason score [n (%)]	
2-6	134 (55.1)
7	70 (28.8)
8-10	39 (16.0)
Extracapsular extension [n (%)]	
No	228 (93.8)
Yes	15 (6.2)
Seminal vesicle invasion [n (%)]	
No	232 (95.5)
Yes	11 (4.5)
Positive surgical margin [n (%)]	
No	206 (84.8)
Yes	37 (15.2)

### Construction of tissue microarray (TMA)

To construct tissue microarray for immunohistochemistry, we retrieved tissues paraffin blocks for these prostate cancer and BPH patients from Pathology Department and prepared 4-μM-thick tissue sections and then stained with hematoxylin and eosin (H&E) to confirm diagnosis and identify the representative tissue morphology for tissue microarray construction. Compared to this H&E-stained tissue section, two 2 × 2 mm tissue cores of each case were taken from the corresponding paraffin blocks using a tissue microarray maker and put into receiving paraffin blocks to generate the tissue microarrays. A total of fourteen tissue microarrays were generated with 5 × 10 tissue cores of each and sectioned for 4-μm-thick tissue sections [15, 16].

### Immunohistochemistry

For immunohistochemical analysis of Connexin 43 expression, the TMA sections were deparaffinized in xylene and rehydrated in a series of ethanol solutions. The TMA sections were subjected to antigen retrieval in 0.1 M citric acid buffer (pH5.0; Fuzhou Maixin Biotech. Co., Ltd., Fuzhou, China) using a high-pressure cook and then subjected to incubation in 3% H_2_O_2_ for 10 min to block endogenous peroxidase activity. The sections were washed with phosphate buffered saline (PBS) three times and incubated with 20% normal goat serum at the room temperature for 30 min and further with a monoclonal anti-Connexin 43 antibody (Cell Signaling Technology, Inc.; Danvers, MA, USA) at a dilution of 1:200 at 4°C overnight. After that, the sections were washed with PBS three times and then incubated with a secondary antibody (DAKO company, Carpentaria, CA, USA) at 37°C for 30 min and subsequently with a ChemMate™ EnVision™ Detection Kit (DAKO company). To visualize the primary antibody-binding signal, the sections were stained with 3,3′-diaminobenzidine (DAB) solution and counterstained with hematoxylin briefly and then mounted with a coverslip. Previous confirmed positive sections were used as a positive control, while 20% normal goat serum replaced the primary antibody and used as a negative control. The immunostained TMA sections were reviewed and scored under a light microscope (Olympus, Tokyo, Japan) by two pathologists separately and any discrepancies were resolved by their re-reviewing of the sections. These TMA sections were semi-quantitatively scored for staining intensity and proportionally staining of positive cells. Staining intensity was scored for 0 (no staining), 1 (weak staining), 2 (moderate staining), and 3 (strong staining), while the percent positivity of staining was defined as 0 (<5%), 1 (5% -25%), 2 (26% -50%), 3 (51% -75%), 4 (> 75%). The final Connexin 43 expression score was calculated using the value of percent positivity score multiply staining intensity score as “-” (score, 0-1), “+” (score, 2-3), “++” (score, 4-5) and “+++” (score ≥ 6) [17].

### Statistical analyses

All statistical analyses were performed using SPSS 19.0 statistical software (SPSS, Chicago, IL, USA). Quantitative data were compared using independent samples *t* test, Mann-Whitney U test or Kruskal-Wallis test, while qualitative data were compared using independent sample chi-square test or Fisher's exact test. Kaplan-Meier plots and the log-rank test were performed to assess the association of Connexin 43 expression with biochemical recurrence-free survival (BFS). The univariate and multivariate Cox proportional hazards regression models were used to associate Connexin 43 expression with clinicopathological data and biochemical recurrence. P<0.05 was considered statistically significant.

## RESULTS

### Association of reduced Connexin 43 protein in prostate cancer tissues with clinicopathological data

**Figure 1:**
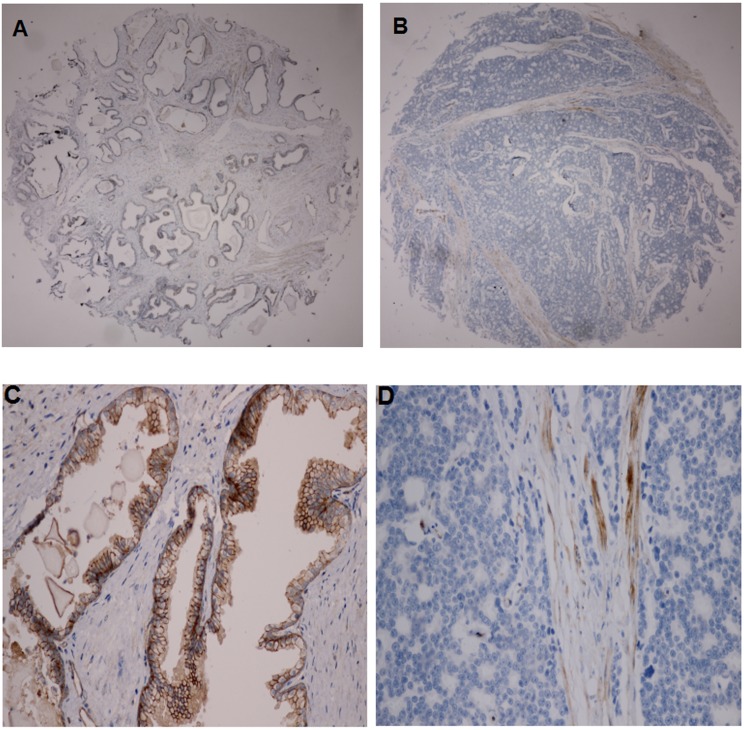
Immunohistochemical staining of Connexin 43 expression in BPH and prostate cancer tissue specimens **A.** and **C.**, high Connexin 43 expression in BPH; **B.** and **D.**, lost Connexin 43 expression in prostate cancer tissues (A/B, ×40; C/D, ×400).

Our immunohistochemical data showed that Connexin 43 protein was mainly expressed in the cytomembrane of prostate epithelia cells in BPH tissues (Figure 1), but lost or reduced in prostate cancer tissues (39.1% *vs*. 96.7%; *P* <0.001).

We then associated Connexin 43 expression with clinicopathological features of these patients (Table 1 and 2). We found that prostate cancer patients had comparable mean age, prostate volume, and BMI to those of BPH patients (*P* > 0.05), whereas the mean Cx43 expression was higher in BPH patients than in PCa patients (*P* < 0.001). The mean PSA level in PCa patients was higher than that in BPH patients (*P* < 0.001; Table 2).

We then associated Connexin 43 expression with clinicopathological data from prostate cancer patients. Our data showed that reduced Connexin 43 expression was associated with higher preoperative PSA levels, Gleason score, and pT stage, positive surgical margin, extracapsular extension, and seminal vesicle invasion (*P* < 0.05, Table 3), whereas Connexin 43 expression was not associated with age of patients, BMI and prostate volume (*P*> 0.05; Table 3).

**Table 2:** Clinicopathological features of the included patients

Variables	PCa	BPH	*p* value
Number of cases	243	60	
Age (years)			
Mean ± SD	68.00 ± 7.04	67.8 ± 7.10	0.85
Range	46 -79	47-81	
BMI (kg/m^2^)			
Mean ± SD	25.42 ± 4.40	25.71 ± 3.90	0.63
Range	17.02 - 33.25	17.06 - 32.98	
PSA level (ng/ml)			
Mean ± SD	13.99 ± 10.21	4.78 ± 3.18	< 0.001
Range	2.02 - 59.10	0.54 - 15.05	
Prostate volume (cm^3^)			
Mean ± SD	38.23 ± 10.21	41.28 ± 15.42	0.07
Range	23 - 57	35 – 145	
Cx43 expression, n(%)			< 0.001
-	148 (60.9)	0	
+	22 (9.1)	0	
++	45 (18.5)	2 (3.3)	
+++	28 (11.5)	58 (96.7)	

### Association of clinicopathological factors and Connexin 43 expression with biochemical recurrence of prostate cancer

Univariate Cox proportional hazards analysis showed that higher pre-operative PSA level, higher Gleason score, advanced pT stage, positive surgical margin, extracapsular extension and seminal vesicle invasion, but lower Connexin 43 expression were all associated with prostate cancer biochemical recurrence after a radical prostatectomy. The multivariate Cox proportional hazards analysis further revealed that higher Gleason score, advanced pT stage, and reduced Connexin 43 expression were all independent predictors for prostate cancer biochemical recurrence after a radical prostatectomy (*P* < 0.05; Table 4).

**Figure 2:**
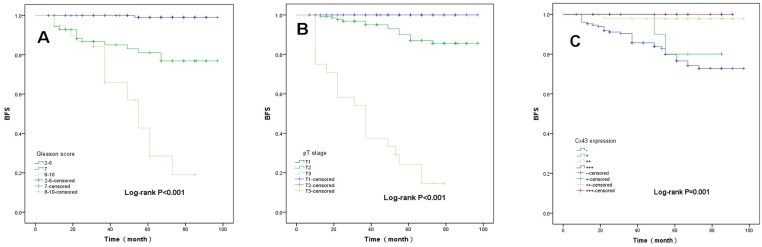
Kaplan-Meier curve analyses of biochemical recurrence-free survival of prostate cancer patients **A.**, Stratified by Gleason score; **B.**, Stratified by tumor pT stage; **C.**, Stratified by Connexin 43 expression.

Kaplan-Meier curve analysis showed that reduced Connexin 43 expression, high Gleason score, and advanced pT stage were associated with shortened BFS of prostate cancer patients after radical prostatectomy (Figure 2).

**Table 3:** Association of Connexin 43 expression with clinicopathological features of prostate cancer patients

Variables	*N*	Connexin 43 expression				*p* value
		-	+	++	+++	
Total, n (%)	243	148 (60.9)	22 (9.1)	45 (18.5)	28 (11.5)	
Age (years)						
< 70	132 (54.3)	74 (50.0)	20 (90.9)	20 (44.4)	18 (64.3)	0.21
≥ 70	111 (45.7)	74 (50.0)	2 (9.1)	25 (55.6)	10 (35.7)	
BMI (kg/m^2^)						
≤ 25	136(56.0)	83 (56.1)	8 (36.4)	19 (42.2)	26 (92.9)	0.29
> 25	107(44.0)	65 (43.9)	14 (63.6)	26 (57.8)	2 (7.1)	
Prostate Volume (ml)						
≤ 35	136(56.0)	92 (62.2)	6(27.3)	22(48.9)	16 (57.1)	0.07
> 35	107(44.0)	56 (37.8)	16 (72.7)	23 (51.1)	12 (42.9)	
PSA level (ng/ml)						
< 10	141 (58.0)	69 (46.6)	5 (22.7)	44 (97.8)	23 (82.1)	<0.001
≥ 10	102 (42.0)	79 (53.4)	17 (77.3)	1 (2.2)	5 (17.9)	
Gleason Score						
2-6	134 (55.1)	67 (45.3)	15 (68.2)	29 (64.4)	23 (82.1)	<0.001
7	70 (28.8)	46 (31.1)	5 (22.7)	14 (31.1)	5 (17.9)	
8-10	39 (16.0)	35 (23.6)	2 (9.1)	2 (4.4)	0 (0)	
pT stage						
T1	89 (36.6)	32 (21.6)	15 (68.2)	25 (55.6)	17 (60.7)	<0.001
T2	130 (53.5)	94 (63.5)	6 (27.3)	19 (42.2)	11 (39.3)	
T3	24 (9.9)	22 (14.9)	1 (4.5)	1 (2.2)	0 (0)	
Extracapsular extension						
Yes	15 (6.2)	14 (9.5)	1 (4.5)	0 (0)	0 (0)	0.007
No	228 (93.8)	134 (90.5)	21 (95.5)	45 (100)	28 (100)	
Seminal vesicle invasion						
Yes	11 (4.5)	10 (6.8)	1 (4.5)	0 (0)	0 (0)	0.028
No	232 (95.5)	138 (93.2)	21 (95.5)	45 (100)	28 (100)	
Positive surgical margin						
Yes	37 (15.2)	28 (18.9)	7 (31.8)	2 (4.4)	0 (0)	0.007
No	206 (84.8)	120 (81.1)	15 (68.2)	43 (95.6)	28 (100)	

**Table 4:** Univariate and multivariate Cox proportional hazards analyses of prostate cancer biochemical recurrence-free survival

Variables	Univariate analysis		Multivariate analysis	
	HR (95% CI)	*P* value	HR (95% CI)	*P* value
Age (years; < 70 *vs*. ≥ 70)	0.82 (0.42-1.62)	NS		
BMI (≤ 25 *vs*. > 25)	1.27 (0.65-2.47)	NS		
Prostate Volume (≤ 35 *vs*. > 35)	0.99 (0.50-1.94)	NS		
Percentage of positive biopsies (< 50% *vs*. ≥ 50%)	1.84 (0.93-3.64)	NS		
PSA level (< 10 *vs*. ≥ 10)	4.00 (1.97-8.11)	<0.001	0.41 (0.13-1.27)	NS
PSAD (< 0.15 *vs*. ≥ 0.15)	0.61 (0.29-1.29)	NS		
Gleason score (2-6 *vs*. 7 *vs*. 8-10)	6.38 (3.80-10.74)	<0.001	6.11 (2.83-13.15)	<0.001
pT stage (T1 *vs*. T2 *vs*. T3)	14.23 (7.50-27.00)	<0.001	8.56(3.57-20.50)	<0.001
Extracapsular extension	9.73 (4.74-19.95)	<0.001	2.20(0.44-10.92)	NS
Seminal vesicle invasion	7.62 (3.45-16.86)	<0.001	0.38(0.92-1.59)	NS
Positive surgical margin	4.66 (2.38-9.11)	<0.001	0.86(0.30-2.42)	NS
Connexin 43 (− / + / ++ / +++)	0.30 (0.14-0.63)	0.002	0.45(0.21-0.99)	0.049

NS, not significant.

## DISCUSSION

Previous data showed that most patients diagnosed with prostate cancer do have a favorable disease progression-free survival after radical prostatectomy, although there is a small proportion of patients facing a risk of tumor metastasis and death [18]. Other studies showed that body mass index (BMI), preoperative prostate-specific antigen (PSA) level, percentage of positive biopsies, pathological T stage, lymph node metastasis, positive surgical margin, extracapsular extension, and seminal vesicle invasion were major risk factors in developing prostate cancer biochemical recurrence [19–22]. Previous studies explored various tumor biomarkers or nomograms to predict prostate cancer biochemical recurrence with different merits [17–21]. Our current study assessed Connexin 43 expression for association with clinicopathological features and biochemical recurrence of prostate cancer after radical prostatectomy. We found that i). Connexin 43 protein was significantly reduced or lost in prostate cancer compared to that of BPH tissues; ii). Downregulated Connexin 43 protein was associated with advanced clinicopathological features; iii). Reduced Connexin 43 expression was associated with shortened postoperative BFS; and iv). Reduced Connexin 43 expression, high Gleason score, and advanced pT stage were all independent predictors for BFS of prostate cancer patients after radical prostatectomy.

Indeed, previous studies demonstrated that the abnormal expression of connexins and loss of gap junctional intercellular communication (GJIC) function were associated with the disease progression of urological tumors [23]. The levels of different connexins were inversely associated with degree of tumor malignancy, whereas upregulated expression level of connexins or restoration of GJIC function inhibited tumor growth and reversed tumor malignant phenotypes [24]. Our current study further confirmed reduced or lost Connexin 43 protein in prostate cancer compared to that of BPH tissues. Normally, Connexin 43 is mainly expressed in the cell membrane of mesenchymal and epithelial cells to form a gap junction (GJ), and in turn to communicate with the adjacent cells [25]. Connexin 43 could suppress tumor cell migration and invasion [10, 11]. Habermann et al. [26] reported that Connexin 43 expression was downregulated in prostate cancer compared to that of BPH, which contributed to tumor de-differentiation and progression. The data reported by Fukushima et al. [27] suggested Connexin 43 as a tumor suppressor gene and transfection of Connexin 43 cDNA into human prostate cancer PC-3 cells increased tumor cell sensitivity to Docetaxel. Increase in Connexin 43 expression promoted tumor cell apoptosis and downregulated Bcl-2 expression [25]. Wang et al. [28] showed that GJ not only acted as the bridge for cell communications, but also influenced the permeability of cytotoxic medicine. Restoration of Connexin 43 expression activated TNF-β receptor signaling to induce apoptosis of prostate cancer cells. Connexin 43 is a protective factor against prostate cancer progression. The underlying mechanism may be accounted for due to loss of Connexin 43 expression and the closing of the gap junction, leading to altered immune surveillance and in turn, tumor cells spreading [12, 25]. In a previous study of 23 normal, 43 BPH and 40 prostate cancer tissues [29], Habermann et al. showed that Connexin 43 expression was upregulated in BPH, but lost in prostate cancer patients. In our current study, we did not include non-pathological prostate tissues due to difficulty in obtaining age-matched tissues and the potential field effect of distant non-pathological tissues. However, our current study does not contradict their data, but further extends their findings of Connexin 43 in prostate pathologies. As BPH is a bengin proliferative condition of prostate gland and Connexin 43 expression may be increased accordingly, whereas prostate cancer is a malignant disease and loss of Connexin 43 expression may be responsible to alter cell growth, and differentiation.

To date, prostate cancer treatment decision is usually based on clinicopathological factors, such as T stage, PSA and Gleason score because these features were associated with prognosis of prostate cancer [30–33]. Among them, Gleason score has been considered as the most effective prognostic indicator [30]. Mithal et al. [31] showed that Gleason score could predicate tumor-specific mortality after radical prostatectomy with a very high accuracy, while tumor pT stage and preoperative PSA level can also be used for prediction of prostate cancer biochemical recurrence [32, 33]. Our current study showed that reduced Connexin 43 expression together with high Gleason score and advanced pT stage is an independent predictor for BFS of prostate cancer patients after radical prostatectomy. Thus, future study will evaluated whether Connexin 43 expression can be used as a biomarker for prostate cancer treatment selection, treatment response, and prognosis.

However, the current study may also have limitations due to the retrospective nature of tissue samples and a single protein analysis (alteration of other connexins could also play a role in prostate cancer). In conclusion, our data demonstrated that Connexin 43 expression was reduced or lost in prostate cancer tissues, which was significantly associated with unfavorable clinicopathological parameters and BFS of patients. Thus, more aggressively adjuvant therapy could be needed to treat those patients with lost Connexin 43 expressed prostate cancer.
